# The interpretation of code status concept among pediatric health care workers, a multicenter cross sectional study across Lebanon

**DOI:** 10.3389/fmed.2025.1532724

**Published:** 2025-03-20

**Authors:** Raymonda Chahrour, Amani Bannout, Marianne Majdalani, Rana Yamout, Ali Ismail, Elma Abou Raffoul, Jihane Moukhaiber

**Affiliations:** ^1^Department of Pediatrics and Adolescent Medicine, American University of Beirut Medical Center, Beirut, Lebanon; ^2^Department of Internal Medicine, Palliative Care Program, American University of Beirut Medical Center, Beirut, Lebanon; ^3^Department of Mathematics, Lebanese University, Beirut, Lebanon; ^4^Department of Pediatrics and Adolescent Medicine, Palliative Care Program, American University of Beirut Medical Center, Beirut, Lebanon

**Keywords:** cardiopulmonary resuscitation, end-of-life, resuscitation orders, healthcare worker, pediatrics, palliative care

## Abstract

**Background:**

Cardiopulmonary resuscitation (CPR) use with no considerations given to patient selection or therapeutic aim resulted in extension of the agony, pain and dying process for terminally ill patients. Four Resuscitation-limiting Codes other than Full Code exist. In a conservative country like Lebanon, several factors can influence such decisions, namely the ethical, legal, religious perspectives, pediatric population, and more importantly the lack of protocol, healthcare workers (HCWs) knowledge, understanding and readiness to discuss terminal care with the parents. The objectives of the study are to evaluate the knowledge, behavior and comfort level of Lebanese pediatric HCWs in code status discussions, and to determine major obstacles encountered.

**Methods:**

This is a cross-sectional observational study. An anonymous questionnaire has been sent electronically for 400 pediatric HCWs from different hospitals across Lebanon, over a period of 3 months.

**Results:**

Of the 400 pediatric HCWs recruited, 235 completed the survey. 39.9% of medical doctors (MDs), and 62% of registered nurses (RNs) did not know about code status subtypes. Most of the MDs are using the paternalistic approach. There were significant differences between MDs and RNs regarding their point of view toward code status, but both thought that it was not defined in the Lebanese law (86.7% of MDs vs. 87% of RNs), and are not comfortable in such discussions (79% for MDs vs. 84.8% for RNs). The decisions taken by MDs regarding life-sustaining treatments (LSTs) in different resuscitation-limiting codes showed clearly the knowledge gap. Moreover, attendings and trainees differed significantly in their decisions, where the latter seemed more conservative. Pediatric HCWs in Lebanon are facing major obstacles when it comes to code status decisions.

**Conclusion:**

Code status in Lebanon is an immature concept, and pediatric HCWs are challenged with conflicting decisions and obligations when it comes to code status discussions and LSTs. A multidisciplinary approach, with good communication between different members of the medical team would be the best. Addressing the obstacles encountered, and set a clear protocol will not only unify and solidify the HCWs decisions, but will have positive impact and repercussions on the patient care as well.

## Background

CPR is a set of emergency procedures performed during cardiac and/or respiratory arrest to restore their function, by means of chest compressions, artificial respiratory support, medications and electrical shocks (1). Soon after considering CPR as the miracle procedure in 1960 (2), it was offered for all cases of cardiac or respiratory arrests, with no consideration given to patient selection or therapeutic aim. By the late 1960s, articles began appearing in the medical literature describing the agony many terminally ill patients experienced from repeated resuscitations that only prolonged their death (3). By 1973, the American Hospital Association stated that CPR is not indicated in certain clinical conditions with no hope of recovery and where death is expected (4). Since then, the “Do-Not-Resuscitate” order became applicable.

DNR alone does not mean do not treat, nor to provide less care, it is a request not to attempt CPR after cardiac or respiratory arrest (5), while providing all other forms of LSTs such as assisted ventilation, inotropic support, antibiotics, renal replacement therapies, parenteral or enteral nutrition or hydration, extracorporeal membrane oxygenation, as well as selected surgical interventions. Actually four Resuscitation-limiting codes, other than full code exist: DNR; DNR/Do-Not-Intubate (DNI) indicating the choice not to proceed with invasive mechanical ventilation via endotracheal tube or tracheostomy (6); DNR/Do-Not Escalate (DNE) meaning that there will be no advancement in the level of care provided including the initiation of new life-sustaining measures or intensifying the existing ones (7); DNR/Comfort Care only (DNR/CC) applied for individuals who have reached a severe state of illness where certain medical interventions are no longer advantageous or when death is imminent. It refers to palliative care and the use of medications like narcotics and sedatives, to prevent and alleviate symptomatic mental and physical discomfort experienced during the dying process (8). In their variations, these orders support the patients’ or their families’ wishes to prevent unwanted and often unnecessary invasive treatment and procedures at the end-of-life (EOL) (9). Worthy to mention that the parents can revert any code status at any time.

Several factors from the patient’s side as well as from healthcare personnel’s side interfere with code status discussions. From the patient’s perspective, his age, socio-economic status, religion, nationality, culture and education play a significant role. From the medical side, the ethics, legal considerations, religion and most importantly, the knowledge, understanding and readiness of healthcare providers to discuss EOL care with the patients are crucial. Ethically, most European standards regarding DNR are paternalistic, meaning the physician assumes responsibility for EOL care decisions, thereby shielding the patient and his family from the burden of ‘culpability’ toward such decisions (10–12). In contrast, the American approach emphasizes patient autonomy, asserting the right to self-determination. This means that a competent patient can refuse LST, and reject future LSTs through an advance directive, which remain valid even if he becomes incompetent (13–15). Regardless of these approaches, there is no ethical difference between withholding and withdrawing LST (16). Decisions should prioritize the patient’s best interest, recognizing that dying is not an acute event, it is a whole process, where cardiac arrest is just its final event. From the legal perspective, there were attempts to clarify physicians’ obligations and protect the rights of both the patient and the physician as well, establishing specific standards to be implemented in EOL decisions. In fact, currently almost all states have statutes allowing for advance directives and living wills regarding all forms of medical treatment, in addition to proxy or surrogate decision-making statutes (17–19). From the religion perspective, few religions have specific declarations on the legitimacy of the DNR order. Most Christian denominations are supportive of the moral obligation to exclude aggressive medical treatments that delay death and deprives it of its due dignity. The suspension of futile treatments must not involve the withdrawal of therapeutic care (20). The Islamic tradition holds that LST is commended, until death is unavoidable. However, it is acceptable to refuse LST and allow DNR in the case of incurable or terminal illness. Withdrawal of care is still not allowed unless the patient is brain dead (21, 22). All these factors are challenging for the physician in decision making, and sometimes disrupt his comfort zone, leading to avoiding DNR or EOL discussions with the patient or his family. In order to standardize HCW decision-making, and to optimize patient care, the majority of developed countries established advance care planning which are patient-focused systems that formulate the overall treatment plan, based on a dialogue between the patient and his clinician, as well as a well-organized and detailed policies and protocols, to be followed and implemented for every patient upon hospital admission, such as respect guidance for healthcare professionals by the resuscitation council UK (23), American Nurse Association (24) and American Medical Association DNR policy (25). Their impact on reducing patient’s suffering and limiting HCW’s hesitation and confusion were stated in several studies (26–28). Unfortunately, this is not the case in most of the Middle East countries, including Lebanon.

Although characterized by a variety of cultures, Lebanon is religiously a conservative country, and most of the Lebanese physicians adopted the European or American system during their training, which both cannot be applied in Lebanon due to major legal and cultural differences. Additional obstacle to the implementation and the practice of DNR order is the pediatric population itself which presents unique challenges mainly moral distress to the HCWs (29), patient autonomy and informed consent. Moreover, there is a knowledge gap of Lebanese Code of Ethics from one side, and there is no clear statement in the Lebanese Law toward DNR and LST from the other side. In fact, the Lebanese Code of Ethics states that: “If the patient suffers from terminal illness, the physician should alleviate the patient’s physical and mental pains and provide the appropriate treatment to preserve patient’s life. The primary care physician is not allowed to put an end to patient’s life, but on the other hand it would be better to avoid unnecessary invasive and exaggerated procedures that tend to prolong patient’s suffering. It’s extremely important to help the dying patient till the end in a way that respects his/her dignity” (30). It means that as by the Lebanese law, withdrawing treatment or intervention is prohibited, but withholding them is allowed as long as the patient/legal guardians were informed about each LST options, after which they can precise the decision or action to be taken toward each option.

In view of all the above, we hypothesize that healthcare personnel in Lebanon, despite being in encounter with end stage cases on a regular basis, have knowledge gap regarding the Lebanese Code of Ethics, the exact meaning of DNR, and the four types of Code status which will define the action to be taken toward the rest of LSTs. In this cross-sectional study, we are going to determine the knowledge of code status and its subtypes among pediatric HCWs in Lebanon, their comfort level and the obstacles encountered. If we find that our hypothesis is correct, this will implement the need to seriously address these barriers, reduce the medical gaps, and set a clear protocol to be followed for EOL care of pediatric patients, taking into consideration the Lebanese ethical, religious and legal perspectives.

## Methods

This is a multicenter, cross-sectional observational study. A questionnaire has been sent electronically to pediatric healthcare professionals, working at the main hospitals in different Lebanese Governorate (Beirut: American University of Beirut Medical Center (AUBMC), Sacre-Coeur Hospital, Beirut Governmental University Hospital; North: Haykal Hospital, Nini hospital; South: Ragheb Harb Hospital, Hammoud Hospital; Bekaa: Dar Al Amal Hospital) targeting Pediatric attendings, trainees (Fellows + residents) and nurses working in any pediatric unit (Pediatric/Neonatal Intensive Care Units (PICU/NICU), regular floor, cardiology…), excluding Pediatric emergency department as well as HCWs working in adult departments. The formers were excluded because in our practice, the primary care physician is the one who can discuss and take decisions regarding code status of the patient and the following LSTs, even if this discussion happened late such as during an emergency visit. The convenience sampling approach was chosen to reach a wide range of participants over a large geographical area. We aim to determine the knowledge of code status and its subtypes among pediatric HCWs in Lebanon, their comfort level and the obstacles encountered.

### Data collection methods and instruments used

The Institutional Review Board approved this study (SBS-2023-0256) on 22 April 2024, in accordance with the ethical principles of Belmont, 1979. Survey design adhered to the tailored design method (31) to optimize its clarity and minimize item non-response. We relied on a questionnaire formulated by Kruse et al. (32) for their study entitled: “Challenges to code status discussions for pediatric patients” and we added some questions to meet our objectives. We used face validity check with two pediatric intensivists and one palliative care specialist. They confirmed that the questionnaire items are clear and support the study objectives. The next step was conducting a pilot testing, where we asked randomly selected and from different hospitals 25 medical students (Med IV) and 25 nursing students (trainees) to fill out the questionnaire, followed by interviews to explore their understanding and clarity of the questions, any difficulties encountered, the appropriateness of the response options, the suitability of the length of the questionnaire and the time required for its completion. The questionnaire was formulated on Limesurvey, and designed in a way that the participant cannot go back to the previously answered page to minimize information bias. It was distributed to the potential participants via email on May 7th 2024 over a period of 3 months, with two reminders. It was anonymous, voluntary, and its first page consisted of written informed consent. There was no question regarding their affiliation, hospital name, or the region/area for more confidentiality and privacy. The survey consisted of four major sections. The demographic one (including the age, gender, religion, as well as their educational level, current position and years of practice); the medical knowledge part regarding different types of code status, their familiarity, comfort level in discussing such conversations, their knowledge about the statutes of code status in the Lebanese law, the decision maker, timing of these conversations and the change in patient care after defining his code; the attitude part, consisting of their decision and attitude regarding different medications and procedures in each type of code status; the obstacles section indicating major obstacles or difficulties faced by the HCWs regarding the code status in Lebanon. Two questions were open-ended: their knowledge of code status, where they had to list all available options, and their knowledge about the Lebanese law regarding code status. All other questions were closed-ended with response options provided. The survey consisted of 25 questions, and needed 20 min to complete. A total sample size of 196 is adequate to detect a difference of 0.1 in 2-way comparisons of provider role knowledge, assuming a proportion of 50%, with alpha = 0.05 and power = 80%.

### Data management and analysis plan

We used the statistical software SPSS version 29 for data entry, data management and analysis. Statistical significance will be set at *p* < 0.05. Categorical variables presented as counts and percentages. For the open-ended question: “list the code status options,” we categorized the answers into 3 groups: “I do not know” (for those who said I do not know or who gave false answer like code blue, red…); “DNI/DNR” (for those who listed DNI and/or DNR); and “DNI/DNR and others” (for those who added one or more code status options like “do not escalate” and “comfort care”). The second question: “What does Lebanese law state?” was not used for analysis because it was kept unfilled by the majority, and only seven participants wrote their answers (five of them said that it is prohibited to withdraw life sustaining measures, and two of them talked about palliative care). Chi-square test (or Fishers exact test when cell size <5) was used for bivariate analysis between providers and the expected variables to differ.

## Results

Of the 400 eligible and approached HCWs, 235 completed the survey with a response rate of 58.8%. The majority of the participants were above 30 years of age (*N* = 120; 51.1%), female (*N* = 159; 67.7%) and working as pediatric HCWs for more than 3 years (*N* = 130; 55.3%). The participants showed a mix of all the demographic variables and subcategories including the religion, current position and current division (refer to Table 1).

**Table 1:** Demographics and characteristics of health care workers.

		Count	Column *N* %
Age	≤ 30 years	115	48.9%
	> 30 years	120	51.1%
Gender	Male	76	32.3%
	Female	159	67.7%
Religion	Shia	74	31.5%
	Sunni	63	26.8%
	Christian	78	33.2%
	Druze	20	8.5%
Current position	Attending	74	31.5%
	Fellow	14	6.0%
	Resident	55	23.4%
	Nurse	92	39.1%
Duration in the current work	< 3 years	105	44.7%
	> 3 years	130	55.3%
Current division	PICU	25	10.6%
	NICU	48	20.4%
	Hem-Onc	36	15.3%
	General pediatrician	50	21.3%
	Pediatric rotating resident	55	23.4%
	Other	21	8.9%
Total		235	100%

Hem-Onc, hematology-oncology; NICU: neonatal intensive care unit; PICU, pediatric intensive care unit.

The difference in point of view toward code status options between MDs (attendings and trainees) and RNs, their familiarity and comfort level were shown in Table 2. Results showed that 57 (39.9%) of MDs, and 57 (62%) of RNs had “I do not know” as an answer to “list the code status options” question, with only 6 MDs (4.2%) and one RN (1.1%) listed DNI, DNR and at least one more code status option. MDs and RNs had statistically significant differences in their answers regarding the decision maker, their familiarity with code status and EOL, the timing of such discussions and possible subsequent patient care change. Whereas both agreed that the code status is not defined in the Lebanese law, and the majority seemed not comfortable in discussing the code status with the parents.

**Table 2:** Familiarity and point of view toward code status in MDs and RNs.

		MD	RN	Total	*p*-value
		Count (%)	Count (%)	Count (%)	
Duration in the current work	< 3 years	67 (46.9%)	38 (41.3%)	105 (44.7%)	0.242
	> 3 years	76 (53.1%)	54 (58.7%)	130 (55.3%)	
Current division	ICU + Hem-Onc^a^	50 (35.0%)	59 (64.1%)	109 (46.4%)	<0.001
	Others	93 (65.0%)	33 (35.9%)	126 (53.6%)	
Type of code status	I do not know	57 (39.9%)	57 (62.0%)	114 (48.5%)	0.003
	DNI/DNR	80 (55.9%)	34 (37.0%)	114 (48.5%)	
	DNI/DNR and others	6 (4.2%)	1 (1.1%)	7 (3.0%)	
Code status defined in Lebanese law	Yes	19 (13.3%)	12 (13.0%)	31 (13.2%)	0.561
	No	124 (86.7%)	80 (87.0%)	204 (86.8%)	
Decision maker	I do not know	17 (11.9%)	29 (31.5%)	46 (19.6%)	0.001
	Attending	56 (39.2%)	22 (23.9%)	78 (33.2%)	
	Both parents	20 (14.0%)	13 (14.1%)	33 (14.0%)	
	Attending + both parents	50 (35.0%)	28 (30.4%)	78 (33.2%)	
Decision maker in case of disagreement	Do not know	17 (11.9%)	30 (32.6%)	47 (20.0%)	0.001
	Attending	72 (50.3%)	28 (30.4%)	100 (42.6%)	
	Father	48 (33.6%)	31 (33.7%)	79 (33.6%)	
	Mother	6 (4.2%)	3 (3.3%)	9 (3.8%)	
Familiarity	Not familiar	57 (39.9%)	57 (62.0%)	114 (48.5%)	0.001
	Familiar	86 (60.1%)	35 (38.0%)	121 (51.5%)	
Comfort level	Uncomfortable	113 (79.0%)	78 (84.8%)	191 (81.3%)	0.176
	Comfortable	30 (21.0%)	14 (15.2%)	44 (18.7%)	
Timing of code status	Too early	2 (1.4%)	0 (0.0%)	2 (0.9%)	0.014
	At the right time	39 (27.3%)	15 (16.3%)	54 (23.0%)	
	Too late	59 (41.3%)	32 (34.8%)	91 (38.7%)	
	Not applicable	43 (30.1%)	45 (48.9%)	88 (37.4%)	
Familial attitude	Receptive	6 (4.2%)	1 (1.1%)	7 (3.0%)	0.008
	Hesitant	34 (23.8%)	23 (25.0%)	57 (24.3%)	
	Resistant	60 (42.0%)	23 (25.0%)	83 (35.3%)	
	Not applicable	43 (30.1%)	45 (48.9%)	88 (37.4%)	
Code status discussion last 6 months	No	59 (41.3%)	47 (51.1%)	106 (45.1%)	0.090
	Yes	84 (58.7%)	45 (48.9%)	129 (54.9%)	
Patient care change	No	24 (16.8%)	29 (31.5%)	53 (22.6%)	0.007
	Yes	119 (83.2%)	63 (68.5%)	182 (77.4%)	
Lecture attended	None	53 (37.1%)	49 (53.3%)	102 (43.4%)	<0.001
	Division lecture /grand round	6 (4.2%)	0 (0.0%)	6 (2.6%)	
	Round/multidisciplinary meeting	61 (42.7%)	42 (45.7%)	103 (43.8%)	
	All	23 (16.1%)	1 (1.1%)	24 (10.2%)	
Total		143 (60.9%)	92 (39.1%)	235 (100%)	

DNI: do not intubate; DNR: do not resuscitate; ICU + Hem-Onc: intensive care unit + hematology-oncology; MD: medical doctor; RN: registered nurse.

a It includes Pediatric ICU, Neonatal ICU and Hem-Onc.

**Figure 1:**
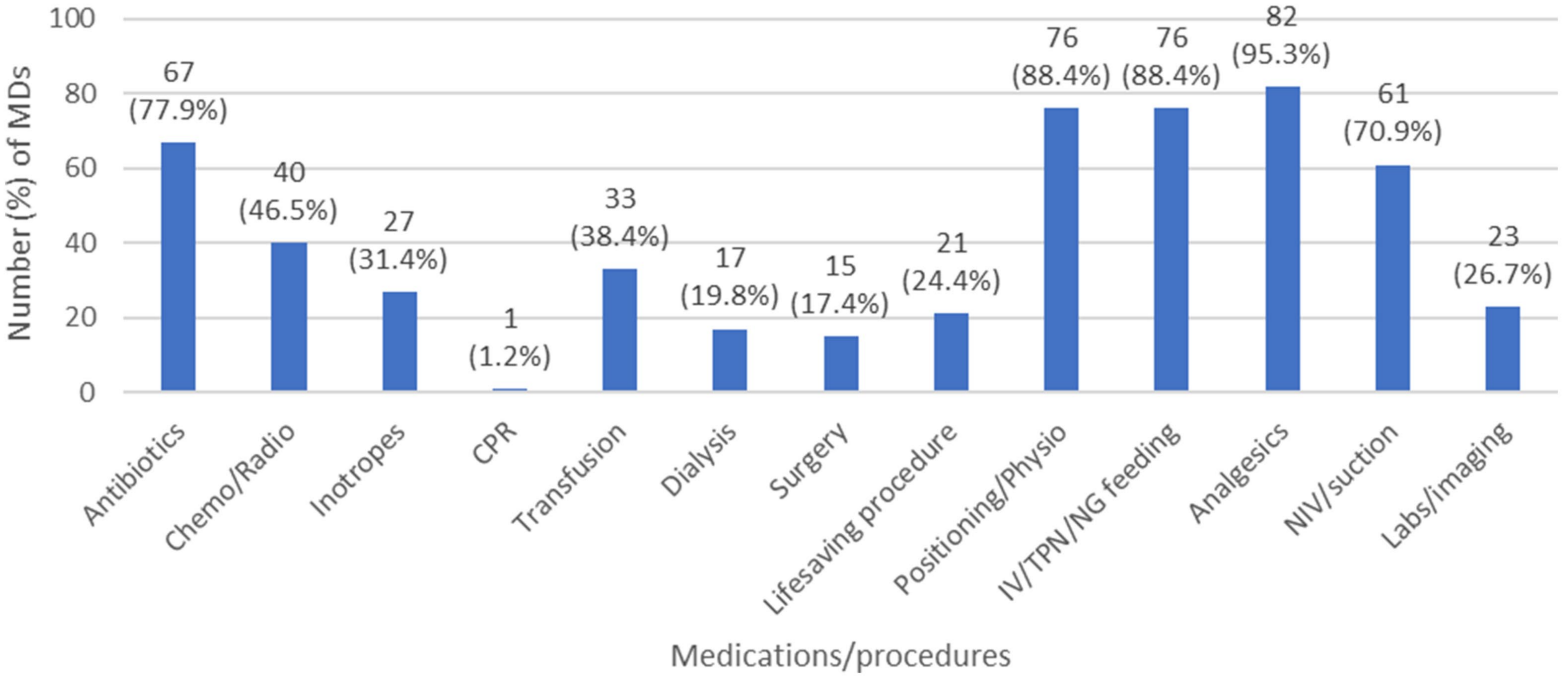
The decisions taken by MDs regarding different medications/procedures in case of DNR. Chemo/Radio, chemotherapy/ratiotherapy; CPR, cardiopulmonary resuscitation; DNR, do not resuscitate; IV/TPN/NG, intravenous/total parenteral nutrition/nasogastric; MD, medical doctor; NIV, non-invasive ventilation; physio, physiotherapy.

**Figure 2:**
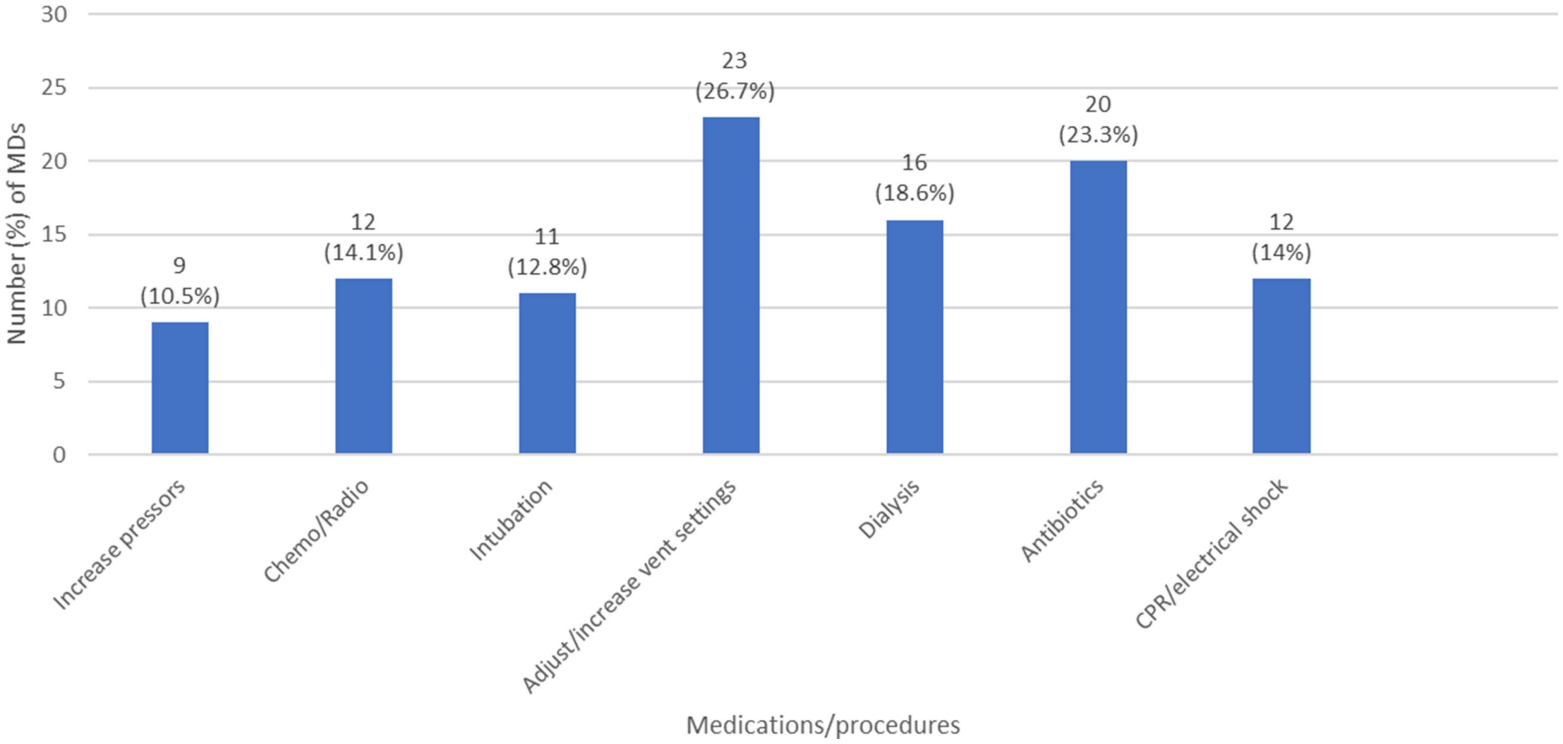
The decisions taken by MDs regarding different medications/procedures in case of DNE. (chemo/radio: chemotherapy/radiotherapy; CPR: cardiopulmonary resuscitation; DNE: do not escalate; MD: medical doctor; vent: ventilator).

**Figure 3:**
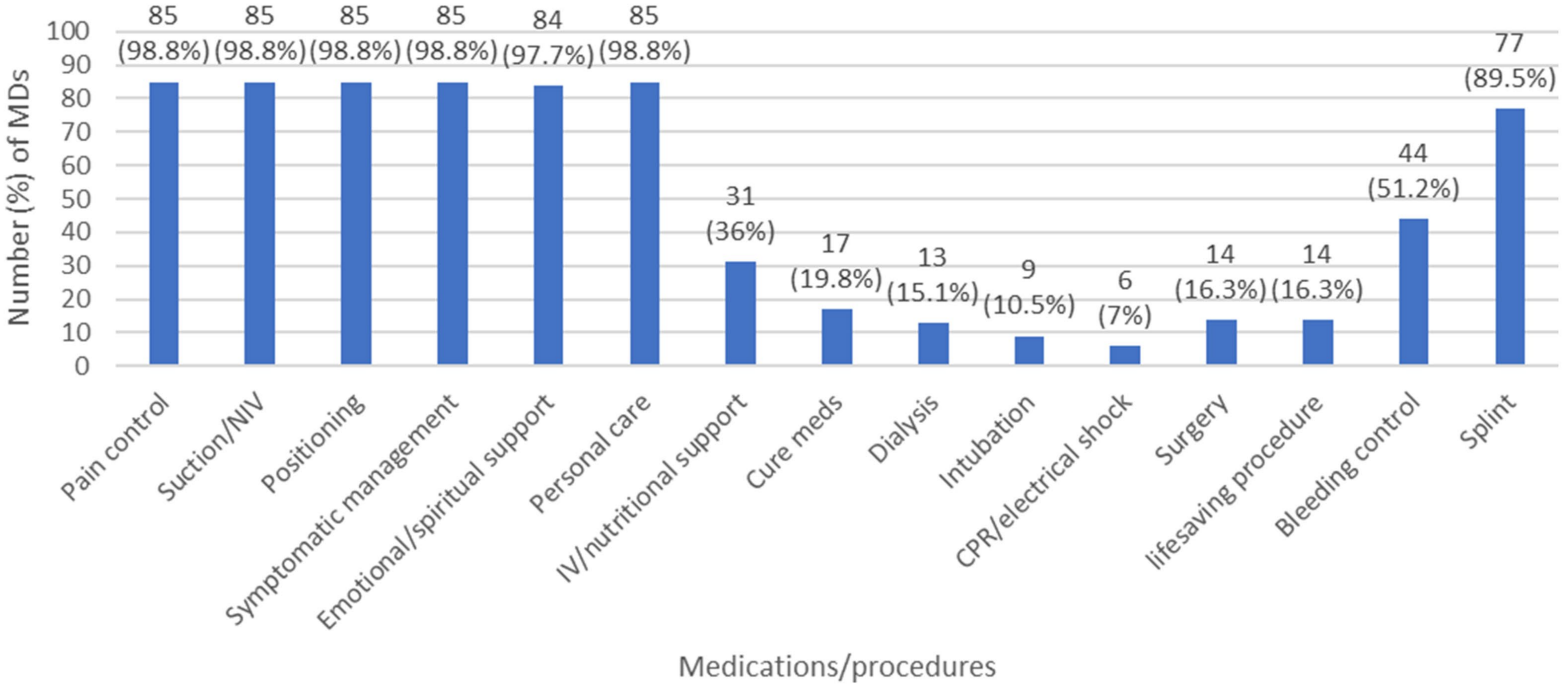
The decisions taken by MDs regarding different medications/procedures in case of Comfort care. (CPR: cardiopulmonary resuscitation; IV: intravenous; MD: medical doctor; NIV: non-invasive ventilation).

In order to take an overview about the decisions taken regarding different medications and or procedures after defining the code status of a patient, we selected the MDs answers, regardless of the RNs answers since such decisions are MD driven. Moreover, to decrease information bias, we excluded MDs who had “I do not know” as an answer to: “list the code status options” question. The results showed that, in case of DNR (Figure 1), MDs tend to limit multiple LSTs including antibiotics, transfusions, inotropes… In case of DNE (Figure 2), 11 (12.8%) MDs choose to intubate the deteriorating patient and 12 (14%) of them will do CPR/electrical shock in case of cardiac arrest. Furthermore, in case of CC (Figure 3), 9 (10.5%) MDs will intubate the deteriorating patient and 6 (7%) of them will perform CPR/electrical shock in case of cardiac arrest.

A secondary analysis was conducted to determine if there is a difference between the attendings and the trainees in the decisions to be taken regarding different code status options. We excluded those who had “I do not know” as an answer to: “list the code status options” question for the same previously mentioned reasons. The results (Table 3) showed that there is a statistically significant difference between the two. The trainees opted to give more medications, or to perform more procedures to the patients than attendings in cases of DNR, DNE as well as CC.

**Table 3:** The difference in answers regarding multiple code status medications and procedures between attendings and residents/fellows.

Code status	Medications / procedures	Attendings / 44	Residents + Fellows /42	*P*-value (95% CI)
		Count (%)	Count (%)	
DNR	Antibiotics	32 (72.7%)	35 (83.3%)	0.178 (−0.279, 0.067)
	Chemo/Radio	20 (45.5%)	20 (47.6%)	0.506 (−0.232, 0.189)
	Inotropes	12 (27.3%)	15 (35.7%)	0.271 (−0.280, 0.111)
	CPR	1 (2.3%)	0 (0.0%)	0.512 (−0.021, 0.066)
	Blood transfusion	13 (29.5%)	20 (47.6%)	0.066 (−0.383, 0.021)
	Dialysis	3 (6.8%)	14 (33.3%)	0.002 (−0.425, −0.104)
	Surgery	3 (6.8%)	12 (28.6%)	0.008 (−0.373, −0.061)
	Lifesaving procedure	7 (15.9%)	14 (33.3%)	0.051 (−0.353, 0.004)
	Physiotherapy, positioning	38 (86.4%)	38 (90.5%)	0.400 (−0.175, 0.093)
	IV fluids, TPN, NG feeding	37 (84.1%)	39 (92.9%)	0.176 (−0.220, 0.045)
	Analgesics	41 (93.2%)	41 (97.6%)	0.326 (−0.131, 0.043)
	NIV, airway suction	30 (68.2%)	31 (73.8%)	0.369 (−0.247, 0.135)
	Labs, imaging	8 (18.2%)	15 (35.7%)	0.055 (−0.359, 0.009)
DNE	Increase pressers	1 (2.3%)	8 (19.0%)	0.012 (−0.294, −0.041)
	Chemo/Radio	3 (6.8%)	9 (21.4%)	0.044 (−0.290, −0.001)
	Intubation	2 (4.5%)	9 (21.4%)	0.020 (−0.307, −0.030)
	Adjust/increase ventilator settings	8 (18.2%)	15 (35.7%)	0.055 (−0.359, 0.009)
	Dialysis	2 (4.5%)	14 (33.3%)	<0.001 (−0.443, −0.132)
	Initiate/escalate Antibiotics	7 (15.9%)	13 (31.0%)	0.081 (−0.327, 0.026)
	CPR/defib/cardioversion	3 (6.8%)	9 (21.4%)	0.049(−0.290, −0.001)
Comfort care	Pain control and sedation	44 (100.0%)	41 (97.6%)	0.488 (−0.022, 0.069)
	Suction, NIV	44 (100.0%)	41 (97.6%)	0.488 (−0.022, 0.069)
	Positioning	44 (100.0%)	41 (97.6%)	0.488 (−0.022, 0.069)
	Symptomatic management (nausea, anxiety)	43 (97.7%)	42 (100.0%)	0.512 (−0.066, 0.021)
	Personal care (hygiene…)	44 (100.0%)	41 (97.6%)	0.488 (−0.022, 0.069)
	Cure meds (Antibiotics, chemo, cardiac drugs…)	5 (11.4%)	12 (28.6%)	0.041 (−0.337, −0.006)
	IV hydration, nutritional support	12 (27.3%)	19 (45.2%)	0.065 (−0.379, 0.020)
	Emotional/spiritual support	43 (97.7%)	41 (97.6%)	0.741 (−0.062, 0.064)
	Dialysis	3 (6.8%)	10 (23.8%)	0.028 (−0.318, −0.021)
	Intubation	3 (6.8%)	6 (14.3%)	0.219 (−0.204, 0.054)
	CPR/defib/cardioversion	1 (2.3%)	5 (11.9%)	0.091 (−0.203, 0.011)
	Surgery	3 (6.8%)	11 (26.2%)	0.015 (−0.346, −0.041)
	Lifesaving procedure	6 (13.6%)	8 (19.0%)	0.349 (−0.210, 0.102)
	Bleeding control	20 (45.5%)	24 (57.1%)	0.193 (−0.326, 0.092)
	Splint	40 (90.9%)	37 (88.1%)	0.470 (−0.101, 0.157)

Chemo: chemotherapy; CI: confidence interval; CPR: cardiopulmonary resuscitation; defib: defibrillation; DNE: do not escalate; DNR: do not resuscitate; IV: intravenous; NG: nasogastric; NIV: non-invasive ventilation; radio: radiotherapy; TPN: total parenteral nutrition.

To note that, despite having a wide demographical distribution as seen in Table 1, that we thought it might influence the results of our study, there was no statistically significant difference in the answers between older age (>30 years) and younger age HCWs, nor between male and female, nor between providers working for more than 3 years in the current work compared to those working for less than 3 years. Moreover, there was no significant difference in the answers between the most exposed HCWs to high acuity patients (NICU + PICU + Hem-Onc attendings) versus other pediatric specialties, and there was no significant difference while comparing the answers of MDs depending on their religion.

**Figure 4:**
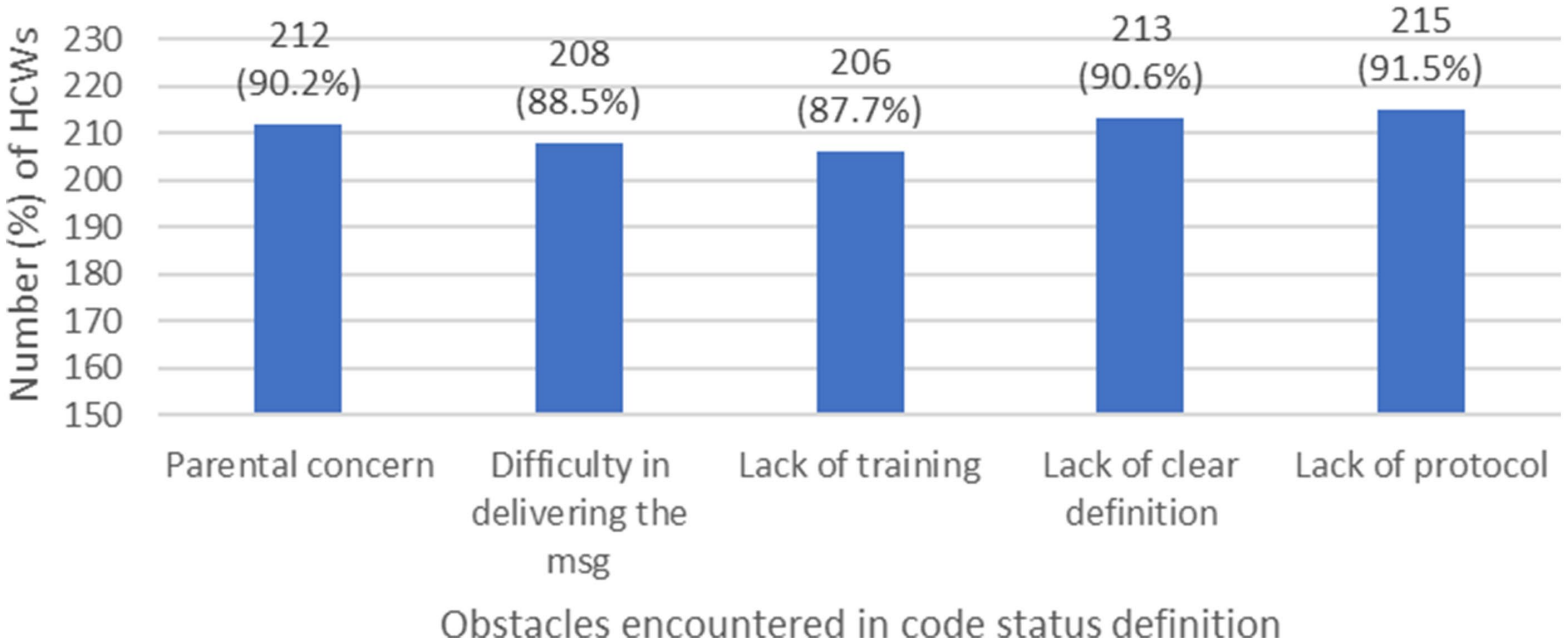
Major obstacles encountered in code status definition by HCWs. (HCW: health care workers, msg: message).

While we saw a discrepancy in understanding the concept of code status between pediatric HCWs, and major differences in practice, the majority agreed that they are facing multiple obstacles in discussing and defining code status in pediatric patients (refer to Figure 4).

## Discussion

Hospital policies and protocols act as the backbone for facility operations (33). They are essential to ensuring a standardized high-quality care for the patients, and guide HCWs in decision taking (34). They help reduce errors, minimize conflicts, maximize agreements between HCWs, while protecting them from internal struggles and guilt feeling, especially when dealing with life-or-death situations and with minors (35). In Lebanon, each hospital has its own policies and protocols, taking into consideration the guidelines as well as its own resources. Unfortunately, this is not the case for code status decisions and the following LSTs, specifically in the pediatric population. To the time of the writing of this article, there are no pediatric studies in Lebanon looking at the knowledge, understanding, and specific LST decisions/actions regarding code status in pediatric HCWs. In this multicenter cross sectional observational study, we used an electronic survey to determine the knowledge and comfort level of HCWs with code status options from different hospitals and different regions, the difference in decisions taken regarding LSTs, as well as the possible obstacles encountered, in order to address these issues, and implement a national protocol that can help pediatric HCWs.

The results showed a scary reality that needs to be addressed urgently. Although MDs were aware of code status options more than do RNs, 114 (48.5%) of the participants did not have any clue about code status concept or options, exceeding by that the knowledge gap mentioned in other countries, (7.3–23.6%) (32, 36). Lack of knowledge is a barrier to quality communication between health care providers as well as with the patient’s family (32), resulting in continuing aggressive therapeutic options, prolonging by that the agony and suffer of the terminally ill pediatric patient, prohibiting them from palliative care and advanced care planning (37, 38), leading to moral distress for the HCWs as well as the patient’s family. Despite that, we did not see any effective measure to overcome this serious knowledge gap on the personal and institutional level. The results showed that overall, 102 (43.4%) were not engaged in any code status and LSTs related lecture/grand round/multidisciplinary meeting/round discussion. This can have serious consequences and repercussions including the avoidance of DNR and EOL discussions, or taking decisions regarding LSTs with hesitation. This can result in delays or inconsistencies in care. Such hesitation can also lead to conflicts among all levels of healthcare providers, as differing perspectives on the appropriateness of interventions may arise. This was apparent in our study, where the majority of HCWs believed that code status discussions occurred too late in the course of disease, whereas 43 (30.1%) of MDs and 45 (48.9%) of RNs had never had such discussions before. Solomon et al. (39) showed that more than two-third of pediatric HCWs think that “they are saving children who should not be saved.”

Code status discussions tend towards paternalistic approach. 56 (39.2%) of MDs said that it is the decision of the attending only, whereas in case of disagreement, the paternalistic approach becomes even more dominant, reaching 72 (50.3%). Although this has been shown in two studies previously conducted in Lebanon, but we can clearly see the trend toward more shared decision between MDs and the parents in comparison to the past behavior. In fact, a study by Sabbagh et al. (40), about the perspective of emergency physicians regarding withholding LSTs in Lebanon showed a paternalistic relationship, where patients and families were involved in the decision-making process in 2.5% only. In 2021, Dabar et al. (41) compared physicians’ approach toward EOL care between the AUBMC and Hotel Dieu de France (which follows the European system), where it showed paternalistic approach in both, but more dominant at Hotel Dieu de France, where it can reach up to 71.9%.

Although there is a statement in the Lebanese law about code status and LSTs as mentioned previously, it is treating this concept in a general superficial way, lacking precision and accuracy. As expected, 204 (86.8%) of MDs and RNs (*p* = 0.561) are unaware of the Lebanese law statement, increasing by that their insecurity and hesitancy feelings. Additional barrier encountered globally, was a widely spread false belief between health care providers at all levels, that patient care will change after code status order, providing less robust basic medical care and less attention (42–45). In our study, 182 (77.4%) of HCWs retained this misconception. This can be explained in part by lack of knowledge, resulting in false practices, which will be passively acquired from medical generation to another, like announcing DNR order, and limiting some forms of LSTs, a practice completely not consistent with the definition of DNR order (5). In another part, cultural, educational, societal, and religious values in Lebanon interfere significantly and have negative influence on such decisions (41).

Although several studies demonstrated that HCWs’ attitude toward code status decisions vary widely amongst religions (46–48), this was not apparent in our study. It could be explained in part by the severe lack of knowledge in our country, irrespective of the religions, and by the parental religion and culture in the other part, which we did not take into consideration in this study. To explain it more, feeling of insecurity, hesitancy and internal struggles by HCWs, will be projected to the parents and patient’s family. The latter will find no convincing or appropriate answers, and feel in need for other more solid resources, hence they will seek religious figures or other self-important person/idol. In 2022, a study done in the PICU at AUBMC by Sabouneh et al. (49) showed that over a period of 5 years (2012–2017), only 34% of families agreed to a DNR order prior to death. Our results showed that 42% of MDs describe familial attitude toward code status discussions as resistant.

A general overview regarding the decisions to be taken by MDs when facing DNR, DNE, or CC orders confirmed the immaturity of these concepts in Lebanon, and the challenges that pediatric HCWs are facing. We can see clearly that in case of DNR, the MDs opted to limit essential LSTs, like antibiotics, inotropes, chemotherapy, radiotherapy, blood transfusions, dialysis… while in DNE and CC, they opted to offer unnecessary LSTs, such as intubation, CPR, electrical shock… raising an important ethical issue to be addressed. The best solution here is to set a clear, national protocol to be followed and implemented for each patient after code status discussions. Most developed countries have established not only code status policies/protocols, but Advanced Care Planning measures as well, during which health care providers discuss in clarity the goals of care, and ensure to the parents what can be offered as a treatment, supportive measures and palliative care, rather than what we will withhold/withdraw after defining the code status (23–25).

The reason behind including all pediatric specialties in our study and not focusing only on NICU/PICU/Hematology-Oncology MDs is that all of them are at risk of being in encounter with code status decisions, like having a pediatric patient with end stage heart failure, or any kind of neurodegenerative disease, or even a previously known DNR patient, admitted to regular floor for comfort care… To our knowledge, there are no previous studies showing familiarity and behavioral differences between pediatric subspecialties, but because logically there is a difference in the frequency of dealing with high acuity patients, we hypothesized that there will be a significant attitude difference between them regarding LSTs in DNR, DNE and CC cases. Surprisingly our study results showed no statistically significant difference when comparing NICU/PICU/Hematology-Oncology MDs in part with all other subspecialties in the other part. Moreover, and for the same previously mentioned reason, we hypothesized that providers that are older in age or having more years of experience, will differ significantly in their attitude compared to the others. Fallahi et al. (50) showed that attitude difference was not found in terms of age group and years of experience, while Khaleghparas et al. (51) showed that HCWs with less than 2 years or more than 20 years of experience demonstrated a more positive attitude toward DNR and Naghshbandi et al. (52) showed positive attitude in HCWs with more than 15 years of experience. Our study rejected this hypothesis. It could be explained by the non-differential severe knowledge gap in code status, inherited from generation to another, irrespective of their age, current division and years of experience.

Provider role is crucial in decision-making. Kruse et al. (32) found that trainees are less comfortable than attendings in discussing code status, and that there were different visions of appropriate care depending on provider’s level. This is consistent with our results, where we had significant differences between attendings and trainees’ decisions. Regardless if this was right or wrong, but the trainees were more conservative about the different LSTs in case of DNR, DNE as well as CC. This highlights the moral stress of the trainees, where they have to abide to what could be considered unethical or inappropriate orders for them.

The obstacles encountered by HCWs have different origin or source, some were at the institutional/national level, which can result in a non-standardized approach toward EOL care of the patients, and inequality in care delivery (overtreatment or undertreatment), others were at the parental/cultural level, confirming that patient’s culture and beliefs contribute to a great extent to the challenges faced by HCWs, and others were at the personal level, which can have serious repercussions on patient care by performing or providing unnecessary procedures and medications, prolonging by that the agony and suffer of the patient, and ignoring his right for palliative treatment which optimize his comfort level and address his concerns carefully. The same obstacles were mentioned in several previous studies (29, 36, 53). However, all these obstacles are interconnected, and one barrier can lead to the other, forming a vicious cycle that needs to be broken. The best way to overcome it, is by starting at the institutional/national level by setting a protocol, and implement it in all the Lebanese hospitals. Next step will be to fill the knowledge gap and increase awareness of code discussions trying to bypass the personal level obstacle. Finally, cultural changes remain the most difficult but not impossible to address. With time, solid HCWs’ knowledge and understanding, we can achieve our goal.

In conclusion, pediatric HCWs in Lebanon are challenged with conflicting decisions and obligations when it comes to code status discussions and LSTs. Their discordant visions are valuable. A multidisciplinary approach, with good communication between different members of the medical team would be the best, because each provider has his own role, approach and support with the patient and his family. Addressing the obstacles encountered, will not only unify and solidify the HCWs decisions, but will have positive impact and repercussions on the patient care as well.

### Strengths and limitations

In this study, we used an electronic questionnaire, to decrease social desirability bias and eliminate interviewer bias. The survey was distributed to HCWs of the main hospitals in different Lebanese Governorate, to be able to get a representative sample of the population in study. Hospitals name was not included in the survey, to preserve the anonymity of the participants. Although the response rate was 58.8%, it has grossly a non-differential selection bias between providers. While the calculated sample size needed was 196, total participant number was 235, increasing by that the power of our analysis.

But our study had some limitations. It focused on the provider’s perspective only, without assessing parental knowledge and point of view toward code status decisions and discussions. The response rate was 58.8%, and we do not know the distribution of answers according to each hospital, this may affect the accuracy of our results (the knowledge and behavior of pediatric HCWs may be different from hospital to another, between the city and rural areas, furthermore there might be a source of selection bias if for some reason providers from a certain hospital did not fill the survey, whereas providers from other hospital participated to some extent). Finally, we included only the biggest hospitals from different locations in Lebanon, with no considerations given to small peripheral hospitals, where the resources are much less, and the code status knowledge could be even worse, leading to ignoring this concept, or on the contrary, to withdrawing/withholding some LSTs without legal consequences.

## Data Availability

The raw data supporting the conclusions of this article will be made available by the authors, without undue reservation.
